# 

**Published:** 1822-10

**Authors:** 


### MONTHLY CATALOGUE OF MEDICAL BOOKS.

An Inquiry into the Action of Mercury on the Living Body. By Joseph Swan, Member of the Royal College of Surgeons, and Surgeon to the Lincoln County Hospital. 8vo. 1s. 6d.

The Seats and Causes of Diseases investigated by Anatomy; containing a great variety of Dissections, accompanied with Remarks. By John Baptist Morgagni. Abridged, and illustrated with copious Notes, by William Cooke, Member of the Royal College of Surgeons, London; and one of the Secretaries to the Hunterian Society. 2 vols. 8vo. 1l. 11s. 6d.

An Essay on the Uterine Hæmorrhage which precedes the Delivery of the full-grown Fœtus; illustrated with Cases. By Edward Rigby, M.D. F.L.S. F.H.S. &c. Sixth Edition, with a Memoir of his Life. 8vo. 7s.

A Catalogue of an extensive Collection of Books, in Anatomy, Medicine, Surgery, Midwifery, Botany, Chemistry, Materia Medica, &c. sold by Callow and Wilson. 12mo. 1s. 6d.

J. Cox's Complete Catalogue of Medical Books and Pamphlets. 8vo. 1s. 6d.

A Treatise on the Utility of Sangui-Suction, or Leech Bleeding; including the Opinions of eminent Practitioners, with Instructions for the Process of Leeching, and an Appendix. By Rees Price, M.D. 12mo. 3s. 6d.

---

### NOTICES TO CORRESPONDENTS.

*We trust that* JUNIUS *will see the propriety of our withholding his last Communication. Having so recently expressed our determination to decline all personal controversies, we cannot be induced to infringe a regulation which we conceive will eventually benefit all, as it will afford more room for the insertion of matter of more general interest. At the same time, we beg to assure* JUNIUS *that we shall feel happy in giving insertion to any Communication with which he may favour us.*

E. T. *will perceive that we have taken some liberties with his paper, but we do not doubt his acquiescence in the propriety of our having done so; and he may, at some future time, thank us for not having recorded his misfortune.*

Dr. K.'*s letter has been received, and the erratum is corrected. We are extremely sorry that it should have escaped our notice when correcting the press: however, it is so obviously a typographical error, that no one for a moment can suspect it to have existed in the manuscript.*

*We return our thanks to* Dr. VON DEM BUSCH, *of Bremen, for his polite letter. The pamphlet to* Mr. SWAN *shall be transmitted; and we can assure the Doctor we shall be most happy to receive any Communication with which he may be inclined to favour us.*

*Numerous Communications have been received: they shall be inserted as soon as possible; but we beg to remind our Correspondents that all original articles must be sent before the 12th of each month, in order to insure their insertion in the following Number.*

*We regret extremely that* Mr. JUKE'*s interesting Communication came too late for insertion in this Number: our readers may rest assured that it shall appear in that for November.*

*The Department of* STATISTICAL MEDICINE *is deferred for want of room.*

